# Blatant Dehumanization of People with Obesity

**DOI:** 10.1002/oby.22460

**Published:** 2019-04-02

**Authors:** Inge Kersbergen, Eric Robinson

**Affiliations:** ^1^ Department of Psychological Sciences University of Liverpool Liverpool UK; ^2^ School of Health and Related Research University of Sheffield Sheffield UK

## Abstract

**Objective:**

Stigmatization of obesity is common, but whether this stigma extends to people with obesity also being considered less human than individuals without obesity has not been examined. This study investigated whether people with obesity are blatantly dehumanized (i.e., explicitly considered to be less human and more animallike) and whether this predicts obesity discrimination.

**Methods:**

In four online studies (total *N* = 1,506) with American, British, and Indian participants, evidence for blatant dehumanization of people with obesity was examined. Whether blatant dehumanization of people with obesity was moderated by BMI and to what extent blatant dehumanization predicted support for weight discrimination were also investigated.

**Results:**

In all studies, participants believed that people with obesity were less evolved and less human than people without obesity. Although blatant dehumanization of people with obesity was most pronounced among thinner participants, the belief that people with obesity were less human was also observed among participants with class I obesity. Finally, dehumanization was predictive of support for policies that discriminate against people living with obesity.

**Conclusions:**

This study provides the first evidence that people with obesity are blatantly dehumanized. This tendency to consider people with obesity as less human reveals the level of obesity stigma and may facilitate and/or justify weight discrimination.

## Introduction

Although obesity is common 1, people with obesity frequently report experiencing mistreatment because of their weight 2, 3. People hold negative attitudes and stereotypes about obesity and treat people with obesity unfairly in various settings 4. Understanding what facilitates obesity discrimination is important as obesity discrimination affects mental and physical health 5, 6. Here, we examine the possibility that prejudiced beliefs about obesity run deeper than previously assumed and that people with obesity are blatantly dehumanized.

Subtle dehumanization has been thought to facilitate prejudice 7, 8. However, a more recently developed concept in social psychology is blatant dehumanization: the overt and explicitly communicated belief that a person(s) is less human than another 9. Blatant dehumanization is theoretically important because explicitly removing a person’s humanity may cause, facilitate, and/or justify mistreatment 9, 10, 11, 12. To date, blatant dehumanization has been studied largely in relation to interracial relations 13, where it is associated with support for policies that result in intergroup conflict 9.

Several considerations underlie our hypothesis that those with obesity may be blatantly dehumanized. Firstly, obesity elicits a disgust response 14, 15, and disgust toward social groups can promote dehumanization 16. Secondly, people with obesity are sometimes portrayed in a dehumanizing manner, displayed as “headless bodies” 17, 18 and described with animalistic language 19. Finally, images of people with obesity are more likely to be implicitly associated with animal‐related words than images of thin people 20.

Across four studies, we examined the blatant dehumanization of those with obesity for the first time. Given the high rates of obesity prejudice reported in the United States 21, we studied US participants before examining generalizability (United Kingdom and India). Besides being stigmatized by people with “normal” weight, people with obesity can themselves be susceptible to antifat bias 22. Therefore, we examined whether blatant dehumanization of obesity is observed among participants with and without obesity. Finally, we examined the relationship between blatant dehumanization of those with obesity and support for policies that discriminate against people with obesity to explore whether blatant dehumanization may play a role in facilitating and/or justifying obesity discrimination.

## Study 1

### Participants

We recruited US members of Amazon Mechanical Turk (MTurk; Seattle, Washington) who had completed at least 100 Human Intelligence Tasks (HITs), with a HIT approval rate of 95% or higher. We planned to examine the difference between humanness ratings of “Americans” and “Obese Americans” using a paired‐samples *t* test. We reasoned that a small to medium effect size (*d*_z_ = 0.30) would be meaningful. A power calculation (G*Power version 3.1; http://www.gpower.hhu.de/) 23 (α = 0.05, 80% power) indicated that we required 90 participants. We recruited slightly more than this to allow for exclusions because of failed attention checks. A total of 111 participants gave consent, and 104 completed the study. Three participants were excluded for failing at least one attention check. The total analysis sample was 101 (Table 1). Excluding one participant who guessed the study aims did not affect the pattern of results. All studies received approval from the University of Liverpool ethics committee.

**Table 1:** Demographic characteristics

	Study 1,	Study 2,	Study 3				Study 4,
	total	total	Total	India	UK	US	total
***N***	101	597	374	107	131	136	434
**Age, mean (SD)**	37.02 (13.38)	37.13 (11.90)	34.78 (10.07)	33.06 (8.14)	36.82 (10.79)	34.18 (10.47)	37.81 (11.22)
**BMI, mean (SD)** a	26.71 (6.35)	27.40 (7.02)	26.19 (7.54)	24.15 (6.78)	26.23 (7.00)	27.64 (8.21)	27.04 (6.39)
**Gender (% female)**	41.60%	52.10%	42.80%	29.90%	55.00%	41.20%	58.30%
**Ethnicity**	87.1%^b^	79.2%^b^	61.5%^b^	96.3%^c^	89.3%^b^	83.1%^b^	82.5%^b^

a Participants with biologically implausible height or weight were excluded from analyses involving BMI: Study 1: *n* = 0; Study 2: *n* = 2; Study 3: *n* = 18 (India: *n* = 9; UK: *n* = 8; US: *n* = 1); Study 4: *n* = 3.

b Percentage white. In Study 3, this variable represents participants identifying as Caucasian.

c Percentage East Asian. There were no Caucasian participants in the sample from India.

### Measures

#### Blatant dehumanization

**Figure 1:**
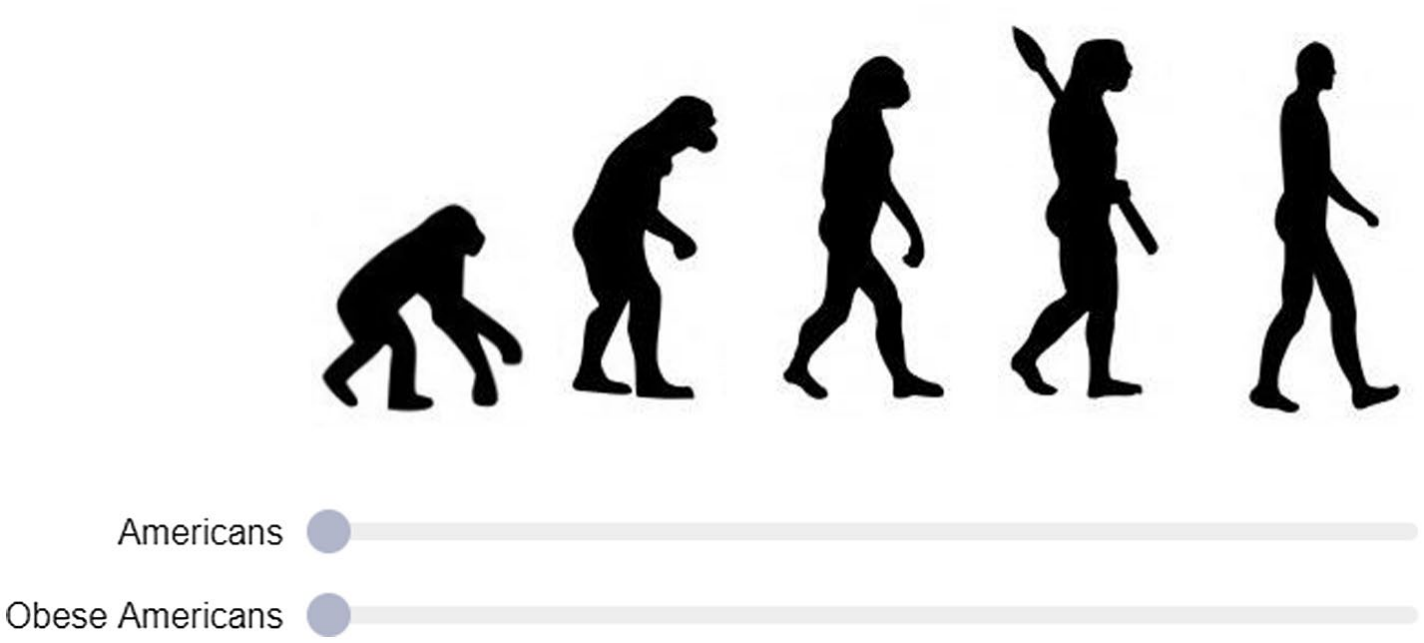
“Ascent of Humans” scale. Participants rated how evolved they consider the average member of several groups to be (in this example “Americans” and “Obese Americans”). Graphic first published in Kteily et al. 9. [Colour figure can be viewed at wileyonlinelibrary.com]

Blatant dehumanization was measured using the Ascent of Humans (AOH) scale 9 (previously named the Ascent of Man scale but renamed to be more inclusive of all humans) 13. The AOH scale is a 100‐point slider scale positioned underneath five ascending silhouettes indicating evolutionary stages between apes and humans (Figure 1). The instructions read, “People can vary in how human‐like they seem. Some people seem highly evolved, whereas others seem no different than lower animals. Using the image below as a guide, indicate using the sliders how evolved you consider the average member of each group to be.” This measure has been used widely to measure blatant dehumanization of social groups 11, 12, 13, 24, 25.

Participants indicated how evolved they considered different groups of people to be (presented in a randomized order). “Americans” and “Obese Americans” were our groups of interest, and “Americans addicted to heroin,” “Arabs,” “Homeless Americans,” and “Employed Americans” were included as filler groups.

#### Other obesity‐related measures

We also included measures of subtle dehumanization of people with obesity, disgust elicited by obesity, weight controllability beliefs, antifat prejudice, and support for weight discriminatory policies to quantify the relationship between each of these measures and blatant dehumanization. See online Supporting Information for full descriptions of these measures and their correlations with dehumanization of those with obesity. Given the lack of research on dehumanization of obesity to date, we also examined whether there was evidence of subtle dehumanization of obesity (Supporting Information).

#### BMI

We calculated BMI from participants’ self‐reported height and weight. Participants whose self‐reported height and weight were outside of the biologically plausible range (height: 1.22‐2.13 m; weight: 34‐227 kg) 26 were excluded from analyses involving BMI.

#### Attention checks

We used attention checks in all studies to ensure that participants were reading the instructions carefully (online Supporting Information).

#### Open data

All analyses in this manuscript were conducted in SPSS Statistics software version 24.0 (IBM Corp., Armonk, New York). Deidentified data and analysis scripts for all studies are available at https://osf.io/qpmxe/.

### Study 1 procedure

After providing informed consent, participants completed a demographics questionnaire (including height and weight), followed by the measures of blatant dehumanization and the other obesity‐related measures (semirandomized order, with support for weight discriminatory policies measured last). Participants then indicated what they thought the aims of the study were before being thanked and debriefed. The survey took approximately 10 minutes, and participants received $0.50 reimbursement.

### Study 1 results

A paired *t* test revealed that “Obese Americans” (mean [*M*] = 78.80, SD = 28.11) were considered to be significantly less evolved and less human than “Americans” (*M* = 88.35, SD = 20.24; *t*[100] = 5.09; *P* < 0.001; *d_z_* = 0.51). For all studies in this manuscript, equivalent nonparametric equivalent tests followed the exact same pattern of statistical significance as the reported parametric tests (online Supporting Information).

## Study 2

In Study 2, we aimed to replicate Study 1 and examine further behavioral evidence of blatant dehumanization. If those with obesity are dehumanized, the likelihood of a person favoring actions that prioritize the reduction of human over animal suffering may be reduced when they believe that those actions will reduce the suffering only of people with obesity.

### Participants

Participants were members of MTurk who had not participated in Study 1. (For all studies in this manuscript, participants could not participate in a study if they had participated in any of the other studies in the manuscript or related pilot studies.) The other eligibility criteria from Study 1 were retained. We planned to examine the difference in proportion of participants donating to the human charity based on condition using a χ^2^ test. We considered a 10‐percentage‐point difference between conditions in the proportion of donations allocated to the human charity to be meaningful. A power calculation (G*Power version 3.1; α = 0.05, 80% power) indicated that we required 588 participants. We recruited slightly more than this to allow for exclusions. A total of 673 participants gave consent, and 635 completed the study. One participant who was not a US resident was excluded, and thirty‐seven participants were excluded for failing the attention check. Total analysis sample was 597 (Table 1). Excluding 24 participants who guessed the study aims did not affect the pattern of results.

### Measures

#### Blatant dehumanization

Blatant dehumanization was measured with the scale used in Study 1, although “Arabs,” “Underweight Americans,” “Americans with a normal weight,” “Americans with cancer,” and “Mixed race Americans” were used as the filler groups.

#### Charity donation

We told participants that we would donate $0.50 to charity to thank them for participating in the study. They could choose between two (bogus) charities: an animal charity (“Action on cruelty to animals”) and a human charity RSM. Dependent on condition, the human charity did or did not explicitly benefit US citizens with obesity: “Our mission is to reduce suffering and mistreatment of [obese] US citizens by campaigning for policy change.” The animal charity was the same in both conditions: “Through raising public awareness we aim to reduce animal suffering in the US.” We measured the proportion of participants who donated to the human charity.

### Study 2 procedure

After providing informed consent, participants provided demographic information (including height and weight), were randomly allocated to one of the charity conditions, and completed the donation task, followed by the AOH scale. Finally, participants indicated what they thought the aims of the study were before being thanked and debriefed. The survey took approximately 2 minutes, and participants received $0.20 reimbursement.

### Study 2 results

#### Blatant dehumanization

A mixed ANOVA with AOH category and charity condition as factors revealed a significant main effect of category, with participants considering “Obese Americans” (*M* = 86.78, SD = 21.91) to be significantly less evolved than “Americans” (*M* = 93.12, SD = 14.16; *F*[1,595] = 70.64; *P* < 0.001; η^2^_p_ = 0.11), replicating the findings of Study 1. As expected, there were no significant effect of condition (*F*[1,595] = 0.21; *P* = 0.64; η^2^_p_ < 0.001) or significant interaction between condition and category (*F*[1,595] = 0.01; *P* = 0.90; η^2^_p_ < 0.001) on the blatant dehumanization measure.

#### Charity donation task

In the “US citizens charity” condition, 39.8% of participants donated to the human charity, whereas only 16.0% of participants in the “obese US citizens charity” condition donated to the human charity. Thus, participants were less likely to favor reducing human over animal suffering when they believed that the human suffering to be reduced was experienced by people with obesity (χ^2^[1, *N* = 597] = 41.66; *P* < 0.001).

## Study 3

In Study 3, we investigated whether the blatant dehumanization of people with obesity would generalize to other cultural contexts. In addition to US participants, we recruited participants from the United Kingdom and India because both of these countries are well represented in online panels. The protocol and analysis strategy of Study 3 were preregistered at https://osf.io/3nrsm/.

### Participants

Participants in this study were members of MTurk living in India who had completed at least 50 HITs, with a HIT approval rate of 95% or higher, and members of Prolific (Oxford, UK) who were residing in the United Kingdom or the United States. We planned to use a similar analysis strategy as in Study 2 and based our power calculation (G*Power version 3.1; α = 0.05, 80% power) on being able to detect a 15‐percentage‐point difference in the proportion of participants donating to the human charity between the two charity conditions (rounded down from the 23‐percentage‐point difference observed in Study 2 to be conservative). We required 326 participants and recruited more than this to allow for exclusions because of failed attention checks. A total of 468 participants gave consent, and 422 completed the study. Nine participants were excluded because of missing data on the donation task, three for reporting a different country of residence than the target countries, and thirty‐six for failing one or more attention checks. The total analysis sample was 374 (India *n* = 107; UK *n* = 131; US *n* = 136; Table 1). Excluding 12 participants who guessed the study aims did not affect the pattern of results.

### Measures

#### Charity donation

As in Study 2, participants were told that we wanted to make a small donation to charity to thank them for taking part. They could choose between two (bogus) charities: an animal charity (“Action on cruelty to animals”) and a human charity CPC. Dependent on condition, the human charity did or did not explicitly benefit people with obesity: “Our mission is to reduce the number of [obese] Indian/UK/US citizens that die young by campaigning for public health policy change.” The animal charity was the same in both conditions: “Through raising public awareness we aim to reduce preventable animal death and inhumane treatment of animals in India/the UK/the US.”

#### Blatant dehumanization

Blatant dehumanization was measured with the AOH scale. Indian and UK participants were asked to rate “[Obese] Indians” and “[Obese] Brits,” respectively. In this study, “Arabs,” “Underweight Indians/Brits/Americans,” “Indians/Brits/Americans with cancer,” and “Mixed race Indians/Brits/Americans” were used as filler groups.

### Study 3 procedure

The procedure was the same as in Study 2. The survey took approximately 2 minutes, and participants received $0.54 reimbursement.

### Study 3 results

#### Blatant dehumanization

**Figure 2:**
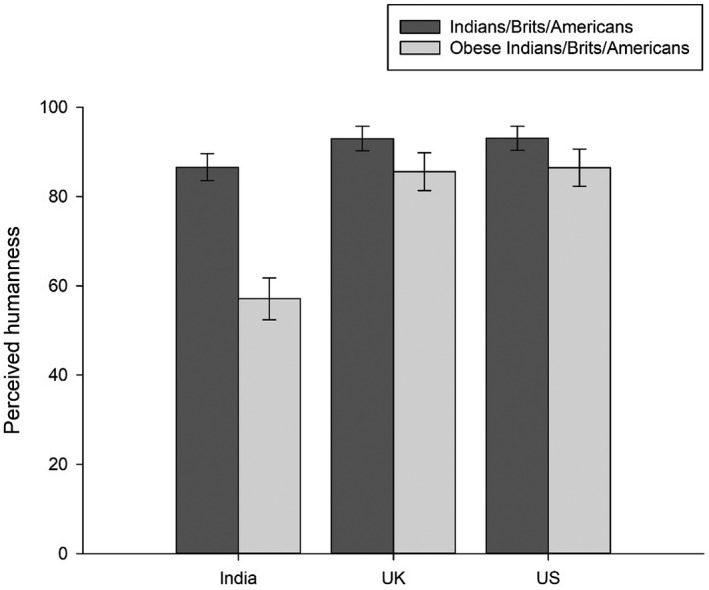
Study 3. Humanness ratings of “[Obese] Indians/Brits/Americans” on the “Ascent of Humans” scale. Higher ratings indicate greater perceived humanness. Bars represent raw means, and error bars represent 95% CIs. All within‐country comparisons were significant at *P* < 0.001.

A repeated‐measures ANOVA with AOH response category as within‐subjects factor and country as between‐subjects factor showed a significant main effect of AOH response category (*F*[1,371] = 169.16; *P* < 0.001; η^2^_p_ = 0.31), a significant main effect of country (*F*[2,371] = 37.88; *P* < 0.001; η^2^_p_ = 0.17), and a significant AOH × country interaction (*F*[2,371] = 42.28; *P* < 0.001; η^2^_p_ = 0.19; Figure 2). Paired‐samples *t *tests showed that “Obese Indians” were rated as significantly less human than “Indians” (*t*[106] = 9.90; *P* < 0.001; *d*_z_ = 0.96), “Obese Brits” as significantly less human than “Brits” (*t*[130] = 4.50; *P* < 0.001; *d*_z_ = 0.39), and “Obese Americans” as significantly less human than “Americans” (*t*[135] = 5.82; *P* < 0.001; *d*_z_ = 0.50).

#### Charity donation task

**Figure 3:**
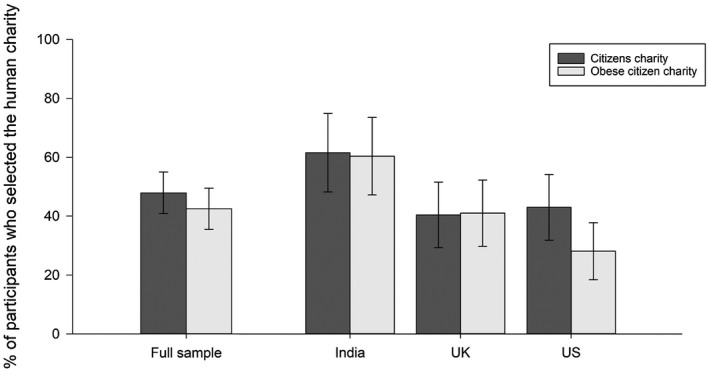
Study 3. Proportion of participants in each country who donated to the human charity, split by charity condition (“citizen charity,” “obese citizen charity”). Error bars represent Jeffreys 95% CIs.

In the “citizens charity” condition, 47.9% of participants donated to the human charity, and 42.5% of participants in the “obese citizens charity” condition donated to the human charity (χ^2^[1, *N* = 374] = 1.10; *P* = 0.29). In a logistic regression accounting for country of residence, there was an overall tendency for participants in the “obese citizens charity” condition to be less likely to favor reducing human over animal suffering, as well as a significant effect of country of residence and no significant interactions between country of residence and charity condition (Table 2). Although the interaction terms between country and condition were not significant, a visual inspection of results (Figure 3) suggests that the nonsignificant tendency for participants to be more likely to donate to the animal versus human charity in the charity‐for‐humans‐with‐obesity conditions was driven by US participants (as observed in Study 2).

**Table 2:** Study 3: logistic regression investigating likelihood of donating to human charity over animal charity predicted by whether the human charity was linked to people with obesity and country of residence

Predictor	Odds ratio^a^	(95% CI)	*P*
**Intercept**	0.28	‐	0.77
**“Obese citizens charity” condition**	0.52	(0.25‐1.05)	0.07
**“Citizens charity” (reference)**	‐	‐	‐
**India**	2.15	(1.03‐4.46)	0.04
**UK**	0.9	(0.45‐1.78)	0.76
**US (reference)**	‐	‐	‐
**India by “obese citizens charity” condition**	1.85	(0.64‐5.32)	0.25
**UK by “obese citizens charity” condition**	1.99	(0.74‐5.40)	0.17
**US by** ** “obese citizens charity” condition (reference)**	‐	‐	‐
**Nagelkerke *R*^2^**	0.07	‐	‐

a Odds ratio represents likelihood of choosing human charity.

## Study 4

In Study 4, we had originally aimed to reduce dehumanization with a media manipulation and examine the effect on discrimination. However, our manipulation was unsuccessful, and we therefore examined the cross‐sectional association between blatant dehumanization and support for discriminatory policies. The protocol and analysis strategy of Study 4 were preregistered at https://osf.io/5h97c/.

### Methods

#### Participants

Participants in this study were US members of MTurk who had completed at least 500 HITs, with a HIT approval rate of 97% or higher. We considered a small to medium effect (*d* = 0.35) of the media manipulation on discrimination to be meaningful. A power calculation (G*Power version 3.1; α = 0.05, 80% power) indicated that we required 384 participants. We recruited more than this to allow for exclusions. A total of 623 participants gave consent, and 481 completed the survey. As specified in the preregistered protocol, 39 participants were excluded for spending less than 10 seconds reading the target article, and an additional 8 participants were excluded for guessing the study aims. The total analysis sample was 434.

#### Experimental manipulation

Participants were randomly allocated to read one of three media articles: an article challenging the idea that dehumanization of people with obesity is acceptable, an article challenging the idea that obesity is under an individual’s personal control (to serve as a control group for exposure to obesity‐related information), and an article unrelated to obesity (discussing the economic importance of tourism). The articles were based on pilot testing and can be found in online Supporting Information.

### Measures

#### Blatant dehumanization

Blatant dehumanization was measured with the AOH scale. We used “Arabs,” “Americans with a normal weight,” “Mixed race Americans,” “Gay Americans,” “Straight Americans,” and “White Americans” as filler groups.

#### Support for weight discriminatory policies

Participants were asked to what extent they would support the following policies in a randomized order: (1) “Increase health insurance premiums for obese people”; (2) “Limit obese people’s access to Medicaid (US government health insurance program for people with low income)”. Support for these policies was indicated on a 9‐point scale (1 = no support at all; 9 = complete support), averaged into a single score.

#### Antifat attitudes

Participants completed the dislike subscale of the Anti‐fat Attitudes scale 27, allowing us to examine whether any effect of blatant dehumanization on support for discriminatory policies would hold when controlling for general dislike of people with obesity.

### Study 4 procedure

We asked participants to participate in two consecutive experiments that ostensibly tested unrelated research questions. In the “first” experiment, participants were exposed to one of the media articles, embedded in a series with two other articles unrelated to obesity (sustainable energy and the Oscars). We informed participants that we were interested in how exposure to media affects mood, and we measured mood before and after each article. In the “second” experiment, participants were told that we were interested in attitudes to various demographic groups. First, participants completed a demographics questionnaire that included measures of race and sexual orientation (to distract from the true aims of the study), as well as height and weight for BMI. Then participants were asked to complete questionnaires about (1) support for weight discrimination and antifat prejudice, (2) racial discrimination and prejudice, (3) discrimination and prejudice based on sexual orientation, and (4) blatant dehumanization. Measures 1 through 4 were displayed in a random order, and the order of the questionnaires assessing prejudice and support for discriminatory policies was also randomized. The questionnaires on discrimination and prejudice based on race and sexual orientation were included to bolster the cover story, and they closely resembled the measures of support for weight discrimination and antifat attitudes. Finally, participants were asked what they thought the aims of the study were with an open‐ended question and a multiple‐choice question before being thanked and debriefed. The study took approximately 10 to 13 minutes, and participants received $1.30 reimbursement.

### Study 4 results

#### Experimental manipulation and awareness of study aims

A one‐way ANOVA showed that humanness ratings of “Obese Americans” did not differ significantly between the three article conditions (*F*[2,431] = 0.92; *P* = 0.40; η^2^_p_ = 0.004). Therefore, the manipulation was unsuccessful. Likewise, a multivariate analysis of variance (MANOVA) showed that article condition did not significantly affect support for discriminatory policies (univariate *F*[2,431] = 0.71; *P* = 0.49; η^2^_p_ = 0.003) or antifat attitudes (univariate *F*[2,431] = 1.20; *P* = 0.30; η^2^_p_ = 0.006). In the multiple‐choice question, 11.3% of participants selected the correct aim of the study. This was not significantly greater than chance expectation (20%). Therefore, we did not examine how correctly identifying the study aims in the multiple‐choice question affected the main results, as specified in the preregistration.

#### Relationship between dehumanization and support for discriminatory policies

We conducted linear regression analysis to explore whether blatant dehumanization of people with obesity was associated with support for discriminatory policies, controlling for experimental condition and antifat attitudes. Blatant dehumanization of obesity was associated with increased support for policies that discriminate against people living with obesity (Table 3).

**Table 3:** Study 4: linear regression investigating association between blatant dehumanization and support for discriminatory policies, controlling for article condition and antifat attitudes

Predictor	*β*	*P*
**Blatant dehumanization** a	−0.10	0.02
**Antifat attitudes**	0.57	*<*0.001
**Article condition (reference: control)**		
**Dehumanization**	−0.05	0.23
**Weight controllability**	0.02	0.70
***R*^2^**	0.37	‐

a Residual difference score of AOH rating of “Obese Americans” predicted by rating of “Americans.” Lower scores indicate greater dehumanization.

#### Supplementary analyses

We pooled data from Studies 1 through 4 to examine whether the level of blatant dehumanization was dependent on participant body weight and to test whether those with obesity were dehumanized to a greater extent than other social groups that deviate from normality based on appearance (underweight Americans) or illness (Americans with cancer). There was a tendency for blatant dehumanization to reduce as BMI increased. Although dehumanization was most pronounced among thinner participants, all BMI groups rated “Obese American” as less evolved and less human than “Americans” except for those with BMI ≥ 35 (online Supporting Information). In addition, “Obese Americans” were rated as being significantly less human and less evolved than “underweight Americans” and “Americans with cancer,” indicating that the dehumanization‐of‐obesity effect is unlikely to be simply caused by people for whom obesity is a social group that differs in physical appearance or has impaired health compared with others. For full analyses and results, see Supporting Information.

## Discussion

Across multiple studies, people with obesity were rated as less evolved and less human than people without obesity. This blatant dehumanization of those with obesity was evident among participants from the United States, the United Kingdom, and India and was associated with greater support for policies that discriminate against people with obesity.

Our results expand on previous literature on obesity stigma by showing that people with obesity are not only disliked and stigmatized 4 but are blatantly dehumanized. Consistent with this, we found some initial evidence among US participants (Studies 2 and 3) that the likelihood of a person favoring actions that prioritize the reduction of human over animal suffering is reduced when they believe that those actions will reduce the suffering of people with obesity. However, there was no evidence that this tendency was observed among UK or Indian participants. Because blatant dehumanization was evident among UK and Indian participants, reduced charitable behavior toward people with obesity may reflect other factors that are unique to the United States rather than blatant dehumanization.

Our results also indicated that blatant dehumanization was most common among thinner participants and was reduced with increasing participant BMI. Participants medically defined as having obesity (BMI 30‐34.9) blatantly dehumanized those with obesity, although participants with more extreme obesity (BMI ≥ 35) did not. The tendency for participants with less extreme obesity to blatantly dehumanize those with obesity is consistent with research showing that weight stigma may be internalized 22. This tendency may be explained by system justification theory 28, which posits that people are motivated to view the social systems in which they operate as fair and may devalue their own social group to maintain this belief 29, 30. However, it is also possible that people with less extreme obesity do not see themselves as having obesity 31, and blatant dehumanization instead resembles outgroup prejudice (i.e., only people who do not identify as having obesity dehumanize those with obesity). The lack of dehumanization among participants with more extreme obesity, who would be more likely to identify as having obesity, is consistent with this proposition, but further research to address this would be valuable.

In a final study, we were unable to reduce the blatant dehumanization of people with obesity and therefore could not examine its causal effect on obesity discrimination. We instead examined the cross‐sectional relationship between dehumanization and support for discriminatory policies against people with obesity. Experimental research showed a bidirectional relationship between dehumanization and interpersonal violence 32. Therefore, blatant dehumanization could plausibly be a cause of obesity discrimination or a way by which obesity discrimination is justified. Further work investigating the causal relationship between blatant dehumanization and obesity discrimination would now be informative.

One strength of this research is that we found consistent evidence across four different studies, increasing our confidence in the reliability of results. We also showed that dehumanization was associated with discrimination after controlling for antifat attitudes, which suggests that dehumanization is distinct from mere dislike. A limitation is that all studies were cross‐sectional and we cannot draw causal conclusions about the influence of dehumanization on discrimination.

Our findings give rise to further unanswered questions that should be addressed in future research. Firstly, Indian participants blatantly dehumanized people with obesity to a greater extent than UK and US participants. The prevalence of obesity is lower in India than in the United States and the United Kingdom 1, suggesting that blatant dehumanization of people with obesity may be greater in cultures in which obesity is less prevalent. However, this difference may also be in part due to cross‐cultural variations in responses to the AOH scale. Secondly, the present studies did not address why obesity is blatantly dehumanized. In previous research, blatant dehumanization was associated with right‐wing authoritarianism and social dominance 9, factors that were also shown to be related to antifat prejudice 33. Future research to understand the individual characteristics that predict whether an individual blatantly dehumanizes people with obesity is now warranted. Finally, information debunking dehumanization of those with obesity or highlighting the complex factors leading to obesity failed to reduce blatant dehumanization of those with obesity. It is possible that the article debunking dehumanization communicated the norm that most people dehumanize obesity, which may have legitimized dehumanizing attitudes 34. Other approaches are required to reduce dehumanization. For example, disgust‐eliciting media portrayals can increase racial outgroup dehumanization 35, and common media portrayals of people with obesity, such as headless bodies 18, may similarly promote blatant dehumanization. Understanding whether contrasting approaches (e.g., pairing obesity with humanizing images) can reduce dehumanization may be valuable.

## Conclusion

The present studies show that people with obesity are blatantly dehumanized and highlight the level of stigma attached to living with obesity in our current societal climate. The tendency to consider people with obesity as being less human than others may facilitate and/or justify discriminatory actions against people with obesity.
