# Supplementary material for: Blatant Dehumanization of People with Obesity
Source: Obesity (Silver Spring). 2019 Apr 2;27(6):1005–12. doi: 10.1002/oby.22460 (PMC6563065; doi:10.1002/oby.22460)
Supplement: Supplementary file 1 [file OBY-27-1005-s001.docx]

**Supplementary materials for ‘Blatant dehumanization of people with obesity’, Kersbergen & Robinson**

Correspondence: Inge Kersbergen, School of Health and Related Research, 30 Regent Street, Sheffield, S1 4DA
Email: [i.kersbergen@sheffield.ac.uk](mailto:i.kersbergen@sheffield.ac.uk)

Contents

[Supplementary measures Study 1 2](#_Toc528060288)

[Supplementary results Study 1 4](#_Toc528060289)

[Non-parametric tests of blatant dehumanization 8](#_Toc528060290)

[Attention checks 9](#_Toc528060291)

[Articles used in Study 4 11](#_Toc528060292)

[Supplementary analyses 16](#_Toc528060293)

[Additional references 19](#_Toc528060294)

# Supplementary measures Study 1

**Subtle dehumanization of obesity**

Subtle dehumanization was measured using an emotion attribution task based on previous research (1,2). On two separate survey pages, participants were asked to indicate on a 100 point slider (anchors: not at all well; very well) how well each of the following emotions characterized ‘Americans’ and ‘Obese Americans’ (randomized order): happiness, euphoria, pleasure, and joy (primary positive emotions); sadness, disgust, anger, and fear (primary negative emotions); tenderness, hope, admiration, and love (secondary positive emotions); and remorse, guilt, shame, and resentment (secondary negative emotions). Emotions were based on (2) and presented in a randomized order. Primary emotions are considered universal (experienced by animals and humans), but secondary emotions are considered to be unique to humans. Therefore, significantly lower scores for secondary emotions (but not primary emotions) for ‘Obese Americans’ than ‘Americans’ are indicative of subtle dehumanization of Americans with obesity.

**Disgust elicited by obesity**

Disgust elicited by obesity was measured with a single item (“How disgusted are you with obese people?”) answer on a 9-point scale (1 = not at all disgusted, 9 = extremely disgusted; (3)).

**Weight controllability beliefs**

Attitudes towards the controllability of weight was measured with the Anti-fat Attitudes questionnaire’s willpower subscale (4). This is a three-item scale answered on a 10-point Likert scale (0 = very strongly disagree; 9 = very strongly agree), with responses averaged into a single score. An example of an item is: “People who weigh too much could lose at least some part of their weight through a little exercise”.

**Anti-fat prejudice**

Anti-fat prejudice was measured with the universal measure of bias towards fat individuals (5). This is a 20-item scale answered on a 7-point Likert scale (1 = Strongly agree; 7 = Strongly disagree), recoded so higher scores indicate greater anti-fat prejudice, averaged into a single score. An example of an item is: “I like fat people”.

**Support for weight discriminatory policies**

Participants were asked to what extend they would support the following three policies in a randomized order: 1) ‘Increase the employment rate of obese people to counter trends in employment discrimination (i.e., affirmative action)’; 2) ‘Limit obese people’s access to Medicaid (US government health insurance program for people with low income)’; 3) ‘More support and training of professionals working to provide legal support for anti-obesity discrimination cases’. Support for these policies was indicated on a 9-point scale (1 = no support at all; 9 = complete support). Policies 1 and 3 were reverse coded and responses were averaged into a single score.

# Supplementary results Study 1

**Correlations**

To calculate correlations, we first summarized blatant dehumanization and subtle dehumanization into single variables. We calculated residual difference scores for rating for ‘Obese Americans’ on the AOH scale (predicted by ratings for ‘Americans’) as a measure of the degree to which participants blatantly dehumanized Americans with obesity, with lower scores indicating relatively lower humanness ratings for ‘Obese Americans’ compared to ‘Americans’ (i.e., greater dehumanization). We calculated residual difference scores for secondary emotion attributions for ‘Obese Americans’ (predicted by secondary emotions attributed to ‘Americans’) as a measure of the degree to which participants subtly dehumanized Americans with obesity, with lower scores indicating that relatively fewer secondary emotions were attributed to ‘Obese Americans’ compared to ‘Americans’ (i.e., greater dehumanization). Pearson’s correlations between measures of blatant and subtle dehumanization, disgust elicited by obesity, weight controllability beliefs, anti-fat prejudice and support for weight discriminatory policies are shown in Table S1.

| **Table S1.** Study 1. Pearson’s correlations between measures of dehumanization and weight stigma. | | | | | | |
| --- | --- | --- | --- | --- | --- | --- |
|  | *M* (SD) | 2. | 3. | 4. | 5. | 6. |
| 1. Blatant dehumanization | .00 (18.84) | -.03 | -.50*** | -.32** | .19^+^ | -.25* |
| 1. Subtle dehumanization. | .00 (14.57) | . | -.06 | -.09 | -.10 | -.11 |
| 1. Disgust elicited by obesity | 3.64 (2.28) |  | . | .41*** | -.10 | .40*** |
| 1. Weight controllability beliefs | 7.35 (1.87) |  |  | . | .02 | .51*** |
| 1. Anti-fat prejudice | 3.94 (1.29) |  |  |  | . | -.08 |
| 1. Support for weight discriminatory policies | 4.57 (1.89) |  |  |  |  | . |
| *Note.* Blatant dehumanization and subtle dehumanization are residual difference scores, with higher scores indicating a lower degree of dehumanization. Therefore, negative correlations between these measures and measures 3-6 indicate positive relationships. *N* = 101. *^+^ p* < .10*,* * *p* < .05, ** *p* < .01, *** *p* < .001. | | | | | | |

**Subtle dehumanization**

A 2 (valence; positive, negative) x 2 (emotion level; primary, secondary) x 2 (group; ‘Americans’, ‘Obese Americans’) repeated measures ANOVA revealed a significant group x level (*F*(1,100) = 19.82, *p* < .001, η^2^_p_ = .17) and group x valence interactions (*F*(1,100) = 33.57, *p* < .001, η^2^_p_ = .25), which were subsumed under a group x level x valence three-way interaction (*F*(1,100) = 16.55, *p* < .001, η^2^_p_ = .14). Mean ratings are shown in Figure S1.

Post-hoc paired samples t-tests showed that participants thought that ‘Obese Americans’ were significantly less likely to feel primary positive emotions than ‘Americans’ (*t*(100) = 4.94, *p* < .001, *d*_z_ = 0.49), but experience primary negative emotions to a similar extent (*t*(100) = 1.05, *p* = .30, *d*_z_ = 0.10). Regarding secondary emotions, ‘Obese Americans’ were considered to be less likely to feel positive secondary emotions than ‘Americans’ (*t*(100) = 4.23, *p* < .001, *d*_z_ = 0.42), but more likely to feel negative secondary emotions than ‘Americans’ (*t*(100) = 5.02, *p* < .001, *d*_z_ = 0.50). Overall, participants considered ‘Obese Americans’ to experience significantly less primary emotions (regardless of valence) than ‘Americans’ (*t*(100) = 2.70, *p* = .008, *d*_z_ = 0.27), but experience secondary emotions to a similar extent (*t*(100) = 1.19, *p* = .24, *d*_z_ = 0.12).


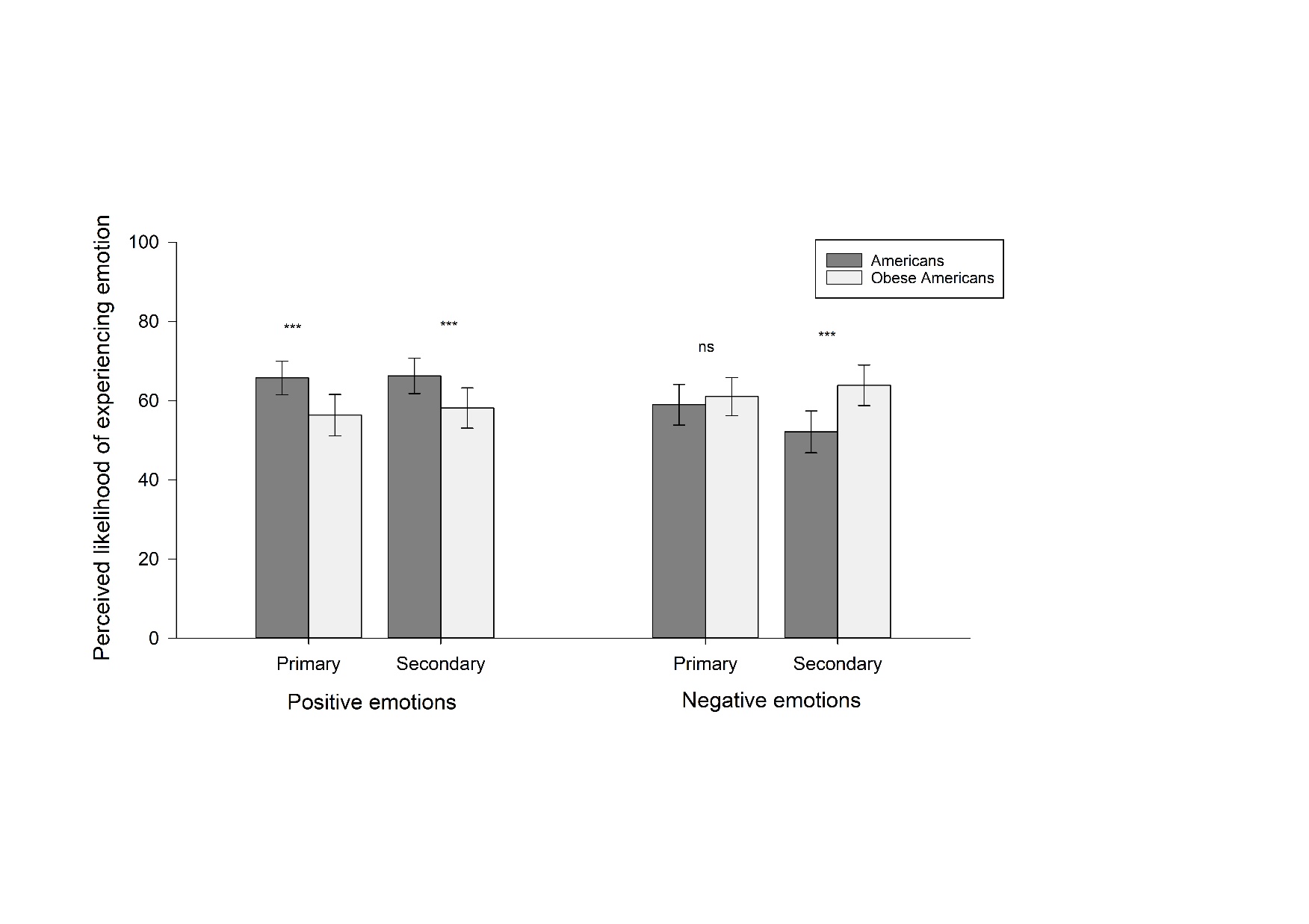


*Figure S1.* Study 1. Extent to which participants attributed primary (universal) and secondary (uniquely human) emotions to ‘Americans’ and ‘Obese Americans’, split by valence. Bars represent raw means and error bars represent 95% CI. *** *p* < .001.

# Non-parametric tests of blatant dehumanization

**Study 1**

A Wilcoxon signed ranks test indicated that ‘Obese Americans’ (Median = 94) were ranked significantly lower than ‘Americans’ (Median = 100; *z* = -5.65, *p* < .001).

**Study 2**

A Wilcoxon signed ranks test indicated that ‘Obese Americans’ (Median = 100) were ranked significantly lower than ‘Americans’ (Median = 100; *z* = -9.50, *p* < .001).

**Study 3**

A Wilcoxon signed ranks tests indicated that ‘Obese Indians’ (Median = 60) were ranked significantly lower than ‘Indians’ (Median = 94; *z* = -7.66, *p* < .001); ‘Obese Brits’ (Median = 100) were ranked significantly lower than ‘Brits’ (Median = 100; *z* = -5.20, p < .001); and ‘Obese Americans’ (Median = 100) were ranked significantly lower than ‘Americans’ (Median = 100; *z* = -5.99, p < .001).

**Study 4**

A Kruskal-Wallis test indicated that ‘Obese Americans’ were not ranked significantly different across article conditions (*H*(2) = 1.35, *p* = .51).

# Attention checks

**Study 1**

We used three attention checks to ensure that participants were reading the instructions carefully: 1) In the measure of blatant dehumanization, we added a slider with the instruction to move the slider all the way to the right; 2) in the measure of support for weight discriminatory policies, we added an item with the instruction to select response option 1; and 3) at the end of the survey, participants indicated what specific group of people was the topic of the study (multiple choice). Participants failed these attention checks if they did not comply with the instructions or did not select “people with obesity” as the group of interest for the study.

**Study 2 and Study 3**

We used one attention check to ensure that participants were reading the instructions carefully. In the measure of blatant dehumanization, we added a slider with the instruction to move the slider all the way to the right. Participants failed this attention checks if they did not comply with the instructions.

**Study 4**

We used eight attention checks to ensure that participants were reading the instructions carefully: 1) In the measure of blatant dehumanization, we added a slider with the instruction to leave the slider all the way on the left; 2) after reading all the articles, participants had to select the topics of the articles in a multiple choice question; 3) in the measure of support for weight discriminatory policies, we added an item with the instruction to select option 6; 4) in the measure of anti-fat prejudice, we added an item with the instruction to select option 1; and 5-8) in the filler questionnaires related to prejudice towards gay people or Arabs we added items with the instruction to select a specific response option. Participants failed these attention checks if they did not comply with the instructions or did not select “obesity” as an article topic. As soon as a participant failed an attention check, the survey was terminated and they were blocked from retaking the survey.

# Articles used in Study 4

**Article challenging dehumanization of obesity**

We need to stop thinking of people with obesity as less human

What do you think when you hear the word ‘obese’? You may come up with some common stereotypes; lazy, greedy, ugly. But researchers from the University of Texas suggest there is more. Their research shows that obesity does not only come with negative connotations, but that people actually think that people with obesity are less human than other people.

The thing that stands out the most from their research is how socially acceptable this dehumanization is. In this research some people openly admitted that they thought that people with obesity were not fully human, and that they would show more respect towards actual animals than people with obesity.

This is perhaps not surprising if you consider how people with obesity are portrayed in the media. They are compared to pigs or whales, and ‘beast’ is a popular play of words on ‘obese’. People with obesity are not only directly compared to animals, the media also portrays them as primitive. Characters with obesity are often shown to have a primitive drive to eat, pushing people out of the way to get to food, or accidentally eating the plate in their hurry to eat.

But that doesn’t mean that it is right! There is no scientific reason to believe that people with obesity are actually less human than other groups and dehumanization is actually very harmful. Dehumanized groups are discriminated against and more likely to face hostilities in everyday life.

Dr Brandt, who led the research, strongly believes that we can no longer think of people with obesity as anything other than human.

**Article challenging personal responsibility of obesity**

Causes of obesity

Modern medical science has gone a long way toward explaining the causes of obesity, and the bottom line is clear: obesity is a result of a complex interplay between someone’s genes and their environment. Whether or not a person becomes obese is in part out of their control; it’s predetermined by their genes and their environment.

Researchers from the University of Texas have shown that the particular set of weight-regulating genes that a person has is an important factor in determining how much that person will weigh. Over the past decade, scientists have identified many of the genes that regulate body weight and have proved that different variants of these genes can lead a person to be obese or thin.

We live in a world where it is easy to find unhealthy fast food meals and harder to find healthy meals. Our lifestyles make it harder to find the time to be physically active and to exercise. A person who is genetically susceptible to eat more or to have a slow metabolism is more likely to become obese in this environment than someone who is not genetically susceptible. This means that obesity is in part caused by a person’s genes and has a lot less to do with the individual than many people commonly believe.

Dr Brandt, who led the research, strongly believes it is important that people fully understand the causes of obesity.

**No obesity information (article about tourism)**

Importance of tourism

Many people love travelling, but tourism doesn’t just benefit individual travelers, but also the local community. Researchers from the University of Texas have shown that tourism is an important factor in the development of local economies.

First you have the money that is spent directly by tourists in the economy, on things like souvenirs, attractions and food. This money earned by local businesses and individual is then re-injected into the local economy. This is why tourism revenue is often referred to as having a multiplier effect, because a large percentage of every tourist dollar earned is reintroduced back into the economy, again and again.

The additional revenue that comes into a community also benefits the local council or governments. More income means more tax revenue, which allows public projects to be launched or developed. The infrastructure improves, with new roads being built, parks developed and public spaces refined. The better facilities bring in more visitors, which supports the economic development even further.

Bringing tourists into a community gives it new life, and creates opportunities for entrepreneurs to establish new services and products, or facilities that would not be sustainable based on the local population of residents alone. Tourists are all potential customers, and with the right approach can be targeted in a business strategy that allows for fantastic success.

Dr Brandt, who led the research, strongly believes that we should encourage tourism in struggling economies.

**Other articles used as part of the cover story**

How Are Oscar Nominees Chosen?

As awards season rolls on, you may be wondering how, exactly, do these Oscar nominations work? Who decides who gets nominated? Well, it’s a complicated process involving lots of math, but here’s what you need to know.

The Academy of Motion Picture Arts and Sciences is made up of around 7,000 filmmakers and film professionals, and these are the people that vote for the Oscars. The organization is divided up into 17 specific branches; a branch for actors, a branch for directors, a branch for editing and so on and so forth.

Nominees for each category are selected by votes from members of these specific branches. For example, only actors get to select nominees for the acting categories, and only directors get to select the nominees for Best Director. However, when it comes to the Best Picture category, everyone gets to vote. For every category, a voting member submits a list of no more than five nominees. But how do these individual ballots turn into the final nominations? This is where the math comes in.

When submitting a list of preferred nominees, Academy members rank them according to preference. The nomination ballots are initially sorted based on the voters’ first-place ranking. If a selection reaches enough first-place votes it becomes a nominee. After that first pass, the stack with the fewest votes is removed, and reassigned according to voters’ second-place selection. The ballots continue to be redistributed in this manner until the minimum number of votes to become a nominee is reached, or until there are only five nominees remaining.

From here, once all the nominations ballots have been counted, new ballots are sent out to every Academy member. For this process, after the nominees have been chosen, everyone gets to vote on every category from the list of nominees chosen by that category’s experts. So while the nomination process is shepherded by experts in every individual category, the final winners are chosen by the esteemed members of the entire Academy.

Blockchain may be the key to a sustainable energy future

The Internet is buzzing this month about the huge potential blockchain technology has to transform industries. Even sectors that seem unlikely, such as the dental industry, are seeing the benefits of this new technology. While Bitcoin has been criticized for being a huge energy consumer, blockchain technology can also help solve our energy problems.

Electricity produced by burning fossil fuels is the number one source of U.S. greenhouse gas emissions. The impacts of burning this dirty fuel are damaging and expensive. Energy access in the developing world is another tremendous problem. As part of its Sustainable Development Goals, the United Nations has a target of providing affordable, reliable and modern energy for all by 2030.

New blockchain solutions are making clean, decentralized energy, such as rooftop solar power, more accessible, affordable and easier to adopt. Blockchain can also enable neighbors to trade clean energy with each other, without needing to go through a utility. It can even power systems that encourage people and businesses to conserve energy.

Blockchain technology is still in its early days, and it’s exciting to ponder the possibilities for the future of energy and the environment. Not only can blockchain redefine today’s broken electricity systems, it can also motivate new levels of climate action and environmental awareness among all of us.

# Supplementary analyses

**Participant BMI**

We excluded all participants with biologically implausible height or weight (*n* = 6). Multilevel regression analysis with Ascent of Humans group (‘Americans’; ‘Obese Americans’) nested within participants and studies (1-4), controlling for participant age and gender showed a main effect of Ascent of Humans group with ‘Obese Americans’ rated as less evolved than ‘Americans’ (*p* < .001). There also was a significant interaction between BMI and AOH response (*p* = .003), with ratings for ‘Obese Americans’ increasing as BMI increased, indicating a reduction in blatant dehumanization as participant BMI increased. Follow-up regressions with BMI split into five categories (< 18.5 ‘underweight’; 18.5-24.9 ‘normal weight’; 25-29.9 ‘overweight’; 30-34.9 ‘obese’; ≥35 ‘severely obese’) showed that although dehumanization was most pronounced among thinner participants, all BMI groups rated ‘Obese Americans’ as less evolved and human than ‘Americans’ (all *p*s < .016), expect those with severe obesity (*p* = .15; Table S2).

| **Table S2.** Pooled analysis. Multilevel regression model investigating the effect of BMI on extent of dehumanization. Ascent of humans (AOH) categories were nested within participants, who were nested within studies. | | | | | | | | | | | | | | |
| --- | --- | --- | --- | --- | --- | --- | --- | --- | --- | --- | --- | --- | --- | --- |
|  | | Total  (*N* = 1262) | |  | BMI < 18.5  (*n* = 43) | | BMI 18.5 - 24.9  (*n* = 526) | | BMI 25 - 29.9  (*n* = 349) | | BMI 30 - 34.9  (*n* = 196) | | BMI > 35  (*n* = 148) | |
|  | | *B* (SE) | [95% CI] |  | *B* (SE) | [95% CI] | *B* (SE) | [95% CI] | *B* (SE) | [95% CI] | *B* (SE) | [95% CI] | *B* (SE) | [95% CI] |
| Fixed effects | | | | | | | | | | | | | | |
|  | Intercept | 88.40 (2.46) | [83.58, 93.23] |  | 98.07 (7.02) | [83.93, 112.20] | 87.91 (2.34) | [83.31, 92.52] | 88.30 (3.12) | [82.17, 94.42] | 83.01 (4.07) | [74.97, 91.04] | 82.99 (4.87) | [73.37, 92.61] |
|  | ‘Obese Americans’ vs ‘Americans’^a^ | -11.16 (1.90) | [-14.88, -7.44] |  | -5.95 (2.37) | [-10.73, -1.18] | -6.85 (0.75) | [-8.33, -5.37] | -6.55 (0.93) | [-8.38, -4.72] | -3.91 (0.86) | [-5.62, -2.21] | -2.02 (1.38) | [-4.75, 0.70] |
|  | BMI | -0.05 (0.07) | [-0.20, 0.09] |  | - |  | - |  | - |  | - |  | - |  |
|  | ‘Obese Americans’ vs ‘Americans’ x BMI | 0.20 (0.07) | [0.07, 0.33] |  | - |  | - |  | - |  | - |  | - |  |
| Random effects | | | | | | | | | | | | | | |
|  | Residual level 2*3 variance (participant*study) | 189.76 (10.62) | [170.04, 211.77] |  | 147.01 (46.55) | [79.04, 273.45] | 181.61 (16.46) | [152.05, 216.92] | 203.62 (21.89) | [164.93, 251.39] | 212.29 (25.40) | [167.91, 268.39] | 151.81 (27.09) | [107.01, 215.38] |
|  | Residual level 1 variance (AOH category) | 136.48 (5.43) | [126.24, 147.56] |  | 120.51 (25.99) | [78.97, 183.91] | 149.43 (9.21) | [132.41, 168.62] | 151.11 (11.44) | 130.27, 175.28] | 73.00 (7.37) | [59.89, 88.98] | 140.71 (16.36) | [112.04, 176.71] |
| *Note:* We controlled for age and gender in all models. ^a^ Scores represent the mean difference in ratings of ‘Obese Americans’ compared to ‘Americans’ on the Ascent of Humans scale. Negative scores indicate lower scores for ‘Obese Americans’ than ‘Americans’. | | | | | | | | | | | | | | |

**Blatant dehumanization compared to other groups**

US participants taking part in study 2 and 3 (*N* = 733) were asked to rate ‘underweight Americans’ and ‘Americans with cancer’ on the AOH scale. Separate multilevel regression models showed that ‘Obese Americans’ were rated as significantly less human than ‘Underweight Americans’ (*B* = -3.39, SE = 0.66, 95% CI = [-4.68, -2.10], *p* < .001) and significantly less human than ‘Americans with cancer’ (*B* = -4.85, SE = 0.64, 95% CI = [-6.10, -3.59], *p* < .001). See table S3 for means and 95% CIs.

| **Table S3.** Human-ness ratings of ‘Obese Americans’, ‘Underweight Americans’ and ‘Americans with cancer’ on the Ascent of humans (AOH) scale. Pooled across studies 2 and 3 (*N* = 733). | |
| --- | --- |
|  | Humanness rating |
| AOH category | *M* (SD) |
| Obese Americans | 86.72 (21.85) |
| Underweight Americans | 90.11 (19.38) |
| Americans with cancer | 91.57 (17.80) |

# Additional references

1. Kteily N, Bruneau E, Waytz A, Cotterill S. The ascent of man: Theoretical and empirical evidence for blatant dehumanization. J Pers Soc Psychol [Internet]. 2015;109(5):901–31.

2. Ehaji SČ, Brown R, González R, Čehajic S. What do I Care? Perceived Ingroup Responsibility and Dehumanization as Predictors of Empathy Felt for the Victim Group. Gr Process Intergr Relations [Internet]. 2009;12(126):715–29.

3. Vartanian LR. Disgust and perceived control in attitudes toward obese people. Int J Obes [Internet]. 2010;34(8):1302–7.

4. Crandall CS. Prejudice against fat people: ideology and self-interest. J Pers Soc Psychol. 1994;66(5):882–94.

5. Latner JD, O’Brien K, Durso L, Brinkman L, Macdonald T. Weighing obesity stigma: The relative strength of different forms of bias. Int J Obes [Internet]. 2008;32(7):1145.
